# Early Childhood Caries and Immunonutritional Status in Preschool Children: A Retrospective Comparative Study of the HALP Score and Hematological Biomarkers

**DOI:** 10.3390/jcm15156122

**Published:** 2026-08-06

**Authors:** Peris Celikel, Yesim Yaptırmıs, Elif Bilici, Hacer Umay Ugurlu, Erol Emir Ceylan, Fuat Cetinkaya, Fatih Sengul

**Affiliations:** 1 Department of Pediatric Dentistry, Faculty of Dentistry, Ataturk University, Erzurum 25040, Turkey; yesimaksu@atauni.edu.tr (Y.Y.); fsengul@atauni.edu.tr (F.S.); 2 Department of Periodontology, Faculty of Dentistry, Ataturk University, Erzurum 25040, Turkey; ebilici82@gmail.com (E.B.); umay_ugurlu@hotmail.com (H.U.U.); eeceylann@gmail.com (E.E.C.); fctnky7@gmail.com (F.C.)

**Keywords:** dental caries, biomarkers, albumins, hemoglobin, child, pediatric dentistry

## Abstract

**Background**: Early childhood caries (ECC) is a common chronic disease in children and has been associated with systemic inflammatory and nutritional alterations. The hemoglobin, albumin, lymphocyte, and platelet (HALP) score reflects immunonutritional status. This study investigated the associations of the HALP score and hematological parameters with ECC, caries burden, and periapical disease severity. **Methods**: This retrospective comparative study included 126 preschool children: 97 with ECC and 29 caries-free controls. Participants were further classified into low (Periapical Index (PAI) 1–3) and high (PAI 4–5) periapical disease severity groups according to the highest Periapical Index score. The HALP score was calculated using the standard formula. Statistical significance was set at *p* < 0.05. **Results**: No statistically significant association was detected between the HALP score and ECC, dmft, or periapical disease severity (*p* > 0.05). Albumin levels were higher in the ECC group than in controls (*p* < 0.001). Neither the HALP score nor its individual hematological components were associated with caries burden in negative binomial regression analyses (*p* > 0.05). Greater caries burden was independently associated with severe periapical disease (Odds Ratio (OR) = 1.19, 95% Confidence Interval (CI): 1.09–1.30, *p* < 0.001), whereas hemoglobin was not (*p* = 0.052). **Conclusions**: No statistically significant association was detected between the HALP score and ECC status, caries burden, or periapical disease severity. Although albumin levels differed between children with ECC and caries-free controls, neither the HALP score nor its individual hematological components were independently associated with caries burden. Larger prospective multicenter studies are needed to validate these findings.

## 1. Introduction

Early childhood caries (ECC) is a major public health problem affecting preschool children worldwide. According to the American Academy of Pediatric Dentistry (AAPD), ECC is defined as the presence of one or more decayed (cavitated or non-cavitated), missing (due to caries), or filled primary tooth surfaces in children from birth through 71 months of age [1]. Because of its high prevalence, early onset, and potential to adversely affect both oral and general health if left untreated, ECC is regarded as a complex disease that remains a major focus of preventive and therapeutic strategies in pediatric dentistry [2]. Early childhood caries remains highly prevalent in Türkiye, particularly in the Eastern Anatolia region, where epidemiological studies have demonstrated a considerable disease burden among preschool children. This high regional prevalence further emphasizes the importance of investigating the potential systemic effects of ECC and identifying clinically accessible hematological biomarkers associated with the disease [3,4].

The etiopathogenesis of ECC is multifactorial, with the structural immaturity of newly erupted primary teeth, extensive colonization by cariogenic bacteria, and an inadequately buffered acidic oral environment considered among its primary determinants [5]. If left untreated, ECC may lead not only to recurrent odontogenic infections, severe dental pain, impaired masticatory function, and esthetic concerns, but also to broader systemic and psychosocial consequences, including impaired growth and development, sleep disturbances, behavioral problems, reduced academic performance, and diminished quality of life. Therefore, ECC should be considered not merely a localized oral disease but a multifaceted clinical condition potentially associated with nutritional status, systemic inflammatory responses, and overall health [6,7].

Chronic dental pain and recurrent odontogenic infections associated with ECC may directly influence children’s dietary habits. Affected children have been reported to avoid hard, fibrous, and protein-rich foods, instead preferring softer foods rich in refined carbohydrates but with lower nutritional value. Such an unbalanced dietary pattern may contribute to deficiencies in protein, vitamins, and minerals, potentially increasing the risk of impaired growth, weight loss, and malnutrition over time [8]. Indeed, children with ECC have been reported to exhibit lower hemoglobin and ferritin levels and a higher prevalence of iron deficiency anemia, suggesting that ECC may serve as a potential risk indicator for unexplained iron deficiency in early childhood. These findings highlight the need to investigate the relationship between ECC and systemic nutritional status using integrated immunonutritional biomarkers [9,10].

In parallel with nutritional alterations, ECC may be associated with systemic inflammatory responses through chronic pulpal and periapical infections. Persistent inflammation has been shown to alter circulating lymphocyte and platelet counts and to modulate immune function. From this perspective, evaluating caries burden and periapical disease severity alongside hematological and immunonutritional markers, rather than relying solely on dental parameters, may provide valuable insights into the systemic implications of oral diseases in pediatric dentistry [11,12].

In recent years, growing interest has focused on practical biomarkers that simultaneously reflect systemic inflammation and nutritional status [13,14]. In this context, the hemoglobin, albumin, lymphocyte, and platelet (HALP) score has emerged as a promising composite biomarker that integrates hemoglobin, albumin, lymphocyte, and platelet levels into a single index, providing a comprehensive reflection of an individual’s immunonutritional status. The HALP score was originally proposed as a prognostic index for patients undergoing surgical treatment for gastric cancer and is calculated using the formula: hemoglobin × albumin × lymphocyte/platelet. Unlike individual hematological parameters, the HALP score combines indicators of nutritional status, immune response, and systemic inflammation into a single composite index. By integrating these complementary biological domains, HALP may provide a more comprehensive assessment of immunonutritional status than isolated laboratory parameters, particularly in conditions where nutritional impairment and chronic inflammation coexist. Since its introduction, the HALP score has been extensively investigated in various malignancies, cardiovascular diseases, and inflammatory conditions, where it has been associated with disease severity and prognosis [15,16].

Although accumulating evidence suggests that ECC may be associated with malnutrition, iron deficiency, and inflammatory processes, studies evaluating these alterations using a composite immunonutritional index such as the HALP score remain scarce [17,18,19]. Consequently, it remains unclear whether the HALP score and its constituent hematological biomarkers are associated with ECC status, caries burden, and periapical disease severity.

A better understanding of the relationship between oral diseases and systemic health during early childhood may contribute to the development of more comprehensive preventive and therapeutic strategies in pediatric dentistry. Therefore, identifying readily accessible biomarkers that reflect both oral and systemic conditions may improve risk assessment and support clinical decision-making. Based on this knowledge gap, the present study aimed to evaluate the associations of the HALP score and its constituent hematological parameters with ECC in preschool children. The primary objective was to investigate whether the HALP score was associated with ECC status and periapical disease severity, while the secondary objective was to examine the associations of individual hematological parameters, including hemoglobin, albumin, lymphocyte, and platelet levels, with caries burden and periapical disease severity. The null hypothesis was that no significant differences would be observed in the HALP score or its constituent hematological parameters according to ECC status or periapical disease severity.

## 2. Materials and Methods

### 2.1. Study Design and Ethical Approval

This retrospective comparative study was designed to evaluate the associations between ECC, immunonutritional status, and hematological biomarkers. Demographic, clinical, radiographic, and laboratory data were retrospectively retrieved from archived dental and medical records maintained by the Department of Pediatric Dentistry, Faculty of Dentistry, and the Department of Pediatrics, Faculty of Medicine, Ataturk University. Laboratory parameters analyzed in this study were obtained from routine blood tests performed as part of standard clinical care and were not collected specifically for research purposes.

Ethical approval for this study was obtained from the Non-Interventional Clinical Research Ethics Committee of Ataturk University Faculty of Medicine (Decision No. 40, Meeting No. 9, 31 October 2025). The study was conducted in accordance with the principles of the Declaration of Helsinki.

### 2.2. Sample Size Calculation

An a priori sample size calculation was performed using G*Power version 3.1.9.2 for a two-sided independent sample comparison. Because no previous study had directly evaluated HALP in children with ECC, the calculation was informed by the systemic inflammatory index data reported by Sarac et al. [12]. The standardized effect size calculated from the reported means and standard deviations was approximately 0.55. With an alpha level of 0.05, a statistical power of 80%, and equal group allocation, the minimum required sample size was estimated as approximately 106 participants, with 53 participants per group. The final study included 126 children; however, the achieved group distribution was unequal, comprising 97 children with ECC and 29 caries-free controls.

### 2.3. Participant Selection and Study Groups

In this retrospective comparative study, archived records of children diagnosed with ECC who underwent comprehensive dental treatment under general anesthesia at the Department of Pediatric Dentistry, Faculty of Dentistry, Atatürk University between January 2025 and June 2025 were retrospectively reviewed. Following ethics approval, the archived records were screened, retrieved, and analyzed between November 2025 and January 2026. The control group consisted of 29 caries-free children who attended the Department of Pediatrics, Faculty of Medicine, Atatürk University between January 2025 and June 2025 and met the eligibility criteria. Participants were classified into the ECC and caries-free groups according to the diagnostic criteria for early childhood caries established by the AAPD [20]. The ECC group consisted of 97 children who met the eligibility criteria and were diagnosed with ECC according to the AAPD criteria. The control group consisted of 29 caries-free children who attended the Department of Pediatrics, Faculty of Medicine, Ataturk University, during the same study period. Children in the control group had a dmft score of 0 confirmed by clinical dental examination and had no systemic disease or active infection.

Children in the control group were selected from those who underwent routine age-appropriate health evaluations and had available clinical and laboratory records. The relatively smaller number of participants in the control group reflects the retrospective design of the study and the strict eligibility criteria. Only healthy 4-year-old children with a dmft score of 0, no systemic disease or active infection, and complete clinical, radiographic, and laboratory records were eligible for inclusion, which substantially limited the number of available controls. To minimize the potential influence of age on hematological, biochemical, and dental parameters, only 4-year-old children were included in both groups.

#### 2.3.1. Inclusion Criteria

Children aged 4 years were eligible for the ECC group if they had been diagnosed with ECC according to the AAPD criteria, had undergone comprehensive dental treatment under general anesthesia, had available preoperative complete blood count and serum biochemical test results, and had complete clinical records together with diagnostic-quality periapical radiographs.

Children aged 4 years were eligible for the control group if they had no history of systemic disease, active infection, or dental caries and had available complete blood count and serum biochemical test results obtained during routine pediatric health evaluations.

#### 2.3.2. Exclusion Criteria

Participants were excluded if periapical radiographs were unavailable or of non-diagnostic quality due to radiographic artifacts, had congenital tooth agenesis or other dental anomalies, or had missing laboratory data for any of the parameters required to calculate the HALP score (hemoglobin, albumin, lymphocyte, or platelet count).

Complete blood count and serum albumin measurements for both the case and control groups were obtained from routine laboratory tests performed in the central laboratory of the same institution as part of standard clinical care.

### 2.4. Collection of Clinical and Laboratory Data

Demographic characteristics (age and sex), clinical data (dmft index), preoperative complete blood count and serum albumin measurements, and periapical radiographs of the ECC group were retrospectively retrieved from patient records and the institutional digital archive. Demographic characteristics and laboratory data of the control group were obtained from the archived medical records of children who attended the Department of Pediatrics, Faculty of Medicine, Atatürk University, for routine pediatric health evaluations. The absence of dental caries in the control group (dmft = 0) was confirmed by clinical dental examination.

All clinical dental examinations, including the assessment of the dmft index and confirmation of caries-free status in the control group, were performed by experienced pediatric dentists as part of routine clinical care at the Department of Pediatric Dentistry. Since this was a retrospective study based on archived clinical records, no additional examiner calibration was performed specifically for the clinical examinations.

All clinical, radiographic, and laboratory data were entered into an electronic database for statistical analysis.

The HALP score was calculated according to the following formula [15]:

HALP = Hemoglobin (g/L) × Albumin (g/L) × Lymphocyte (/L) ÷ Platelet (/L)

### 2.5. Periapical Assessment

**Figure 1:**
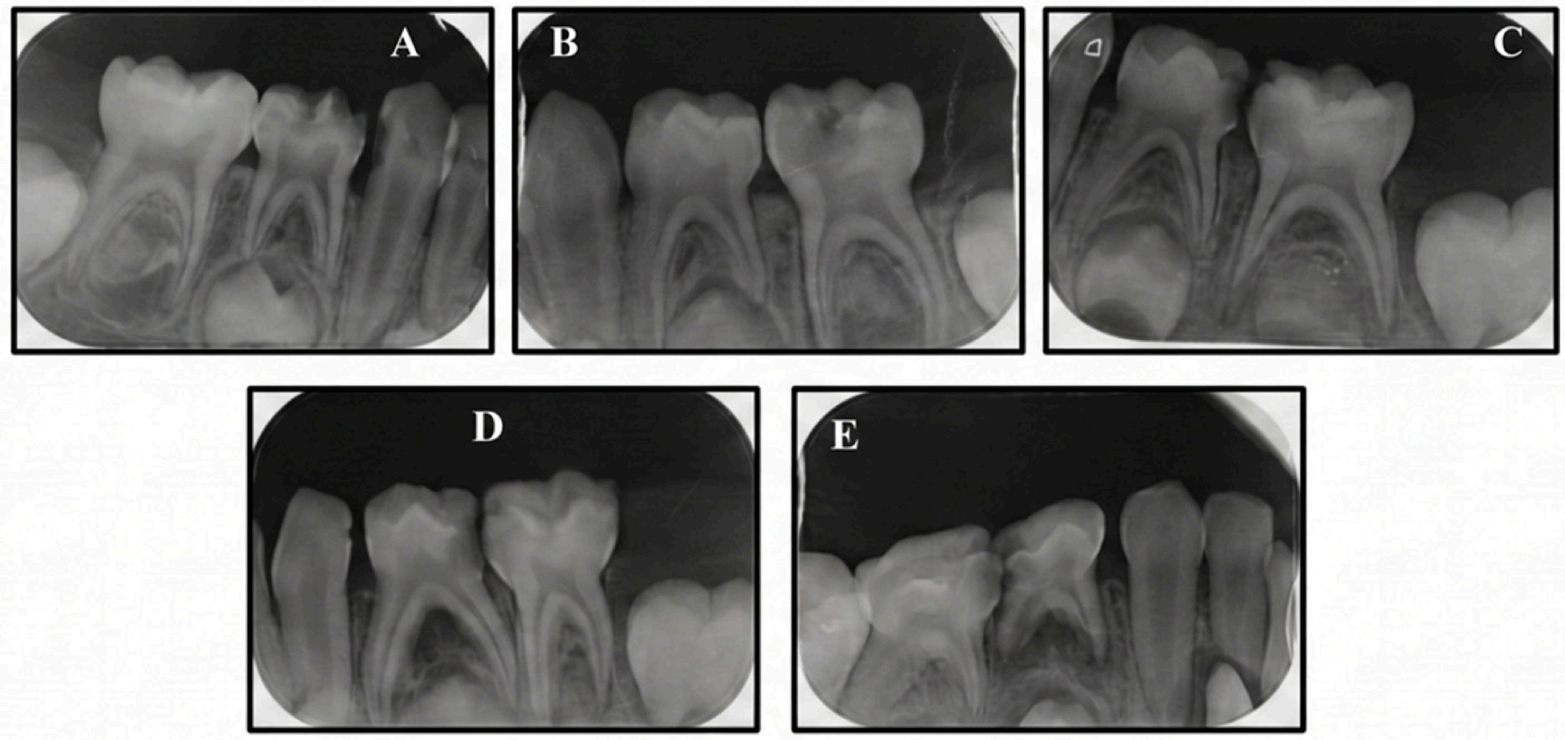
Representative periapical radiographs illustrating the Periapical Index (PAI) scoring system in preschool children with early childhood caries (ECC): (**A**) PAI score 1, (**B**) PAI score 2, (**C**) PAI score 3, (**D**) PAI score 4, and (**E**) PAI score 5.

Periapical radiographs were independently evaluated by two investigators who were blinded to the participants’ clinical and laboratory data. The periapical status was assessed using the Periapical Index (PAI) system described by Ørstavik et al. [21]. This histologically validated scoring system classifies periapical status on a five-point ordinal scale ranging from 1 to 5 according to radiographic findings. The PAI scores were defined as follows:

Score 1: Normal periapical structures (Figure 1A).

Score 2: Small changes in bone structure without evidence of apical periodontitis (Figure 1B).

Score 3: Changes in bone structure with some mineral loss characteristic of apical periodontitis (Figure 1C).

Score 4: Apical periodontitis with a well-defined radiolucent area (Figure 1D).

Score 5: Severe apical periodontitis with exacerbating features (Figure 1E).

For multirooted teeth, the highest PAI score assigned to any root was considered the overall PAI score of that tooth. For participant-level analyses, when more than one tooth was evaluated in the same participant, the highest PAI score was used to represent the individual’s periapical disease severity.

To evaluate the association between periapical disease severity and systemic biomarkers, participants were further categorized according to the highest PAI score recorded for each individual. Children with a highest PAI score of 1–3 were assigned to the low-PAI group, whereas those with a highest PAI score of 4–5 were assigned to the high-PAI group. Because periapical radiographic examination was not clinically indicated in asymptomatic caries-free children, participants in the control group were classified as having normal periapical status (PAI = 1) for group classification purposes.

### 2.6. Calibration

To assess inter- and intra-examiner reliability in the radiographic evaluation of periapical status, two investigators independently evaluated the periapical radiographs. Before the evaluation, both investigators reviewed the PAI scoring criteria described by Ørstavik et al. [21], and a calibration exercise was conducted using 30 randomly selected periapical radiographs. To account for the ordinal nature of the PAI scale, weighted Cohen’s kappa was used. Inter-examiner agreement demonstrated almost perfect reliability (κ = 0.938, 95% CI: 0.88–0.97). Intra-examiner reliability was re-assessed after two weeks (κ = 0.91 and 0.94 for the first and second examiners, respectively). Any disagreements between the examiners were resolved by consulting a third senior expert to reach a final consensus.

### 2.7. Study Variables

The primary analysis evaluated the association between the HALP score and dental outcomes (ECC status, caries burden (dmft), and periapical disease severity). Secondary analyses examined the associations between the individual hematological components of the HALP score (hemoglobin, albumin, lymphocyte count, and platelet count) and the same dental outcomes.

### 2.8. Statistical Analysis

All statistical analyses were performed using IBM SPSS Statistics for Windows, Version 25.0 (IBM Corp., Armonk, NY, USA). The normality of continuous variables was assessed using the Kolmogorov–Smirnov and Shapiro–Wilk tests. Normally distributed variables were expressed as mean ± standard deviation (SD), whereas non-normally distributed variables were presented as median and interquartile range (IQR). Between-group comparisons were performed using the independent-samples *t*-test, Mann–Whitney U test, chi-square test, or Fisher’s exact test, as appropriate. Spearman correlation analysis was used to assess associations between HALP, hematological parameters, dmft, and PAI scores.

Because dmft is a count variable, negative binomial regression with a log-link function was used. HALP was analyzed in a separate model from hemoglobin, albumin, lymphocyte count, and platelet count to avoid mathematical dependency. Multicollinearity among predictors was assessed using variance inflation factors (VIFs), and no evidence of problematic multicollinearity was observed (all VIFs < 2). The assumption of linearity in the logit for continuous predictors was assessed using the Box–Tidwell procedure, and no significant violations were detected (all interaction terms, *p* > 0.05). These models were applied to the total sample and repeated in the ECC subgroup. Binary logistic regression was used to identify factors associated with high PAI scores (PAI 4–5), including dmft, hematological parameters, and sex. Results were reported as regression coefficients or odds ratios with 95% confidence intervals. Model calibration was assessed using the Hosmer–Lemeshow test. A *p*-value < 0.05 was considered statistically significant.

## 3. Results

### 3.1. Sample Characteristics

A total of 126 children (55 girls and 71 boys) with a mean age of 47.3 ± 6.1 months were included in the study. According to ECC status, participants were classified into a caries-free control group (dmft = 0; n = 29) and an ECC group (n = 97). Based on the highest Periapical Index (PAI) score, participants were further categorized into a low-PAI group (PAI 1–3; n = 88) and a high-PAI group (PAI 4–5; n = 38). Descriptive characteristics of the study participants are presented in Table 1. In the overall study population, the median dmft index was 12 (IQR: 0–20), the median PAI score was 3 (IQR: 1–5), and the median HALP score was 6.47 (IQR: 1.66–21.93).

### 3.2. Associations Between Variables

Spearman correlation coefficients between the HALP score and dental variables are presented in Table 2. The HALP score was not significantly correlated with ECC status, the dmft index, PAI score, or PAI group (*p* > 0.05).

### 3.3. Comparison According to Periapical Disease Severity

Comparisons between the low- and high-PAI groups are presented in Table 3. Among the variables evaluated, only the dmft index was significantly higher in the high-PAI group (Z = 3.53, *p* < 0.001). No significant differences were observed between the groups regarding hemoglobin, albumin, lymphocyte, platelet, or HALP score (*p* > 0.30).

### 3.4. Comparison According to ECC Status

Comparisons between the ECC and caries-free control groups are presented in Table 4. As expected, the dmft index was significantly higher in the ECC group (Z = 8.23, *p* < 0.001). Albumin levels were also significantly higher in the ECC group (*p* < 0.001). Although hemoglobin levels tended to be lower in the ECC group, the difference did not reach statistical significance (*p* = 0.065). No significant differences were observed between the groups with respect to lymphocyte count (*p* = 0.624), platelet count (*p* = 0.505), or HALP score (*p* = 0.982).

### 3.5. Negative Binomial Regression Analysis

To further investigate the association between hematological parameters and dental caries experience, negative binomial regression analyses were performed using dmft as the dependent count variable. In the overall study population, HALP score was not significantly associated with dmft (β = −0.015, 95% CI: −0.093–0.062, Wald χ^2^ = 0.149, *p* = 0.699). Similarly, when the individual hematological parameters were entered into a separate model, hemoglobin (B = 0.045, *p* = 0.696), albumin (B = 0.058, *p* = 0.110), lymphocyte count (β = −0.114, *p* = 0.200), and platelet count (B = 0.001, *p* = 0.440) were not significantly associated with dmft (Table 5).

To assess the robustness of these findings, the analyses were repeated in the ECC subgroup. Consistent with the overall analysis, neither hemoglobin (*p* = 0.949), albumin (*p* = 0.818), lymphocyte count (*p* = 0.897), nor platelet count (*p* = 0.806) showed a significant association with dmft. Likewise, no statistically significant association was detected between the HALP score and dmft in children with ECC (*p* = 0.919, Table 5).

### 3.6. Logistic Regression Analysis

Binary logistic regression analysis was performed to identify independent predictors of severe periapical disease (PAI scores 4–5). The overall model was statistically significant (Omnibus χ^2^ = 24.631, *p* < 0.001), demonstrated good calibration according to the Hosmer–Lemeshow goodness-of-fit test (χ^2^ = 4.382, *p* = 0.821), and correctly classified 70.6% of the cases. Among the evaluated variables, only dmft was independently associated with severe periapical disease (OR = 1.188, 95% CI: 1.087–1.299, *p* < 0.001). Hemoglobin was not significantly associated with severe periapical disease (OR = 0.588, 95% CI: 0.344–1.005, *p* = 0.052), whereas albumin, lymphocyte count, platelet count, and sex were not significantly associated with high PAI scores (all *p* > 0.05). The estimated odds ratio indicates that each 1 g/dL increase in hemoglobin was associated with a 41.2% lower odds of severe periapical disease; however, this association did not reach statistical significance (Table 6).

## 4. Discussion

In this retrospective comparative study, we evaluated the associations between the HALP score, individual hematological parameters, and ECC in preschool children. The principal finding was that no statistically significant association was detected between the HALP score and ECC status, caries burden, or periapical disease severity. Albumin levels were significantly higher in children with ECC than in caries-free controls; however, neither the HALP score nor the individual hematological parameters were independently associated with caries burden in the negative binomial regression analyses. Furthermore, greater caries burden was independently associated with severe periapical disease, whereas hemoglobin did not reach statistical significance in the multivariable logistic regression model. Collectively, these findings suggest that the HALP score was not associated with ECC-related dental outcomes in this study. Accordingly, the null hypothesis regarding the HALP score and its individual hematological components was not rejected.

One of the principal findings of the present study was that no statistically significant association was detected between the HALP score, a proposed composite indicator of immunonutritional status, and either ECC status or periapical disease severity. Although the HALP score integrates hemoglobin, albumin, lymphocyte, and platelet levels into a single composite index, statistically significant associations observed for some of its individual components were not reflected in the composite score. The HALP score was originally developed as a prognostic indicator in patients with gastric cancer and has subsequently been investigated as a biomarker reflecting immunonutritional status in various malignancies, cardiovascular diseases, and chronic inflammatory conditions [22,23]. These conditions are characterized not only by persistent systemic inflammation but also by marked nutritional disturbances, including loss of appetite, inadequate nutritional intake, weight loss, and protein–energy malnutrition. Because ECC may also affect children’s dietary habits through dental pain, impaired mastication, and recurrent odontogenic infections, it was hypothesized that the HALP score might also be altered in this population [24]. However, in the present study, no statistically significant association was detected between the HALP score and ECC status or periapical disease severity. These findings should be interpreted cautiously and require confirmation in larger prospective studies incorporating comprehensive nutritional and inflammatory assessments.

The interpretation of the HALP score should also take into account the way in which the index is calculated. The HALP score is a composite immunonutritional index derived from hemoglobin, albumin, lymphocyte, and platelet levels [22]. Consequently, modest and discordant changes in the individual components may counterbalance one another, preventing a measurable change in the overall HALP score. In the present study, albumin levels were significantly higher in the ECC group, whereas hemoglobin levels were associated only with advanced periapical disease severity. In contrast, no significant differences were observed in lymphocyte or platelet counts. Collectively, these findings may explain why individual hematological parameters demonstrated significant associations with specific clinical outcomes, whereas the composite HALP score did not. This finding suggests that alterations observed in individual hematological biomarkers may not necessarily be reflected in the composite HALP score. In addition, most of the available evidence regarding the clinical utility of the HALP score has been generated in adult populations. Consequently, the applicability of this index to preschool children, whose physiological immune and hematological profiles differ considerably from those of adults, remains to be established [25,26]. Because most available evidence regarding the HALP score has been derived from adult populations, its applicability to preschool children remains uncertain and requires confirmation in future pediatric studies.

Among the components of the HALP score, albumin levels were significantly higher in children with ECC than in caries-free controls. However, this between-group difference was not supported by the negative binomial regression analyses, in which albumin was not significantly associated with caries burden in either the overall sample or the ECC subgroup. Albumin is a well-established marker of nutritional status and a negative acute-phase protein whose serum concentration decreases in response to systemic inflammation. Therefore, reduced serum albumin levels are generally expected in clinical conditions accompanied by chronic inflammation and malnutrition. However, the present findings were inconsistent with this expectation, suggesting that the mechanisms linking ECC and serum albumin concentrations in preschool children may differ from those observed in chronic systemic diseases [27]. Nevertheless, studies evaluating the association between ECC and serum albumin levels in children remain limited, and the available evidence is inconsistent. Megha et al. [28] reported no significant difference in serum albumin levels between children with ECC and caries-free controls, whereas Jha et al. [10] found significantly lower serum albumin concentrations in children with severe ECC. In the present study, albumin levels were significantly higher in the ECC group, suggesting that the association between ECC and serum albumin may be more complex than previously assumed and may be influenced by differences in study populations, disease severity, nutritional status, and sampling characteristics. Furthermore, the fact that the study by Jha et al. [10] included only children with severe ECC (S-ECC) may partly explain the discrepancy between the findings. Importantly, albumin levels in both groups remained within the pediatric reference range, indicating that although the observed difference was statistically significant, its clinical relevance may be limited. However, this finding should be interpreted cautiously. Because the study was retrospective and the case and control groups were recruited from different clinical settings, the observed difference in albumin concentrations may have been influenced by unmeasured factors such as nutritional status, hydration status, socioeconomic characteristics, or differences related to the clinical indication for laboratory testing rather than by ECC itself. Accordingly, the present findings should be interpreted as demonstrating an association rather than a direct biological effect of dental caries on serum albumin concentrations. Moreover, because anthropometric measurements, dietary information, and inflammatory biomarkers were unavailable, serum albumin should not be interpreted as a standalone indicator of overall immunonutritional status in this study. Future prospective studies incorporating comprehensive nutritional assessments together with acute-phase proteins and inflammatory biomarkers are warranted to clarify the biological mechanisms underlying this association.

Although hemoglobin showed a trend toward an association with severe periapical disease, this association did not reach statistical significance. Hemoglobin is one of the most widely used hematological biomarkers for assessing iron metabolism and nutritional status in children. In chronic infections and inflammatory conditions, pro-inflammatory cytokines may suppress erythropoiesis, alter iron metabolism, and promote functional iron deficiency, ultimately leading to reduced hemoglobin concentrations [10,29]. Therefore, the finding that hemoglobin was associated with periapical disease severity rather than ECC status suggests that hemoglobin may more accurately reflect the cumulative systemic effects of chronic odontogenic infection than the presence of dental caries alone. However, previous studies evaluating the association between ECC and hemoglobin levels have reported inconsistent findings. Some studies have reported significantly lower hemoglobin levels and an increased risk of iron deficiency anemia in children with severe early childhood caries (S-ECC) [10,30], whereas others found no significant differences in hemoglobin levels between children with ECC and caries-free controls [9]. Furthermore, a higher prevalence and greater severity of ECC have been reported in children with iron deficiency anemia, supporting a possible association between iron metabolism and the development of dental caries [31]. In the present study, although hemoglobin levels did not differ significantly between the ECC and control groups, lower hemoglobin levels showed a trend toward an association with greater periapical disease severity; however, this relationship did not reach statistical significance in the multivariable logistic regression analysis. These findings suggest that systemic hematological alterations may be influenced not only by the presence of dental caries but also by the severity and chronicity of odontogenic infection. Accordingly, our findings highlight the potential importance of considering radiographic indicators of periapical disease, in addition to caries burden, when evaluating the systemic manifestations of ECC.

Most previous studies investigating the association between early childhood caries and systemic biomarkers have evaluated disease severity solely on the basis of caries indices, such as the dmft index [13,32]. In contrast, the present study evaluated systemic biomarkers not only according to ECC status and caries burden but also according to periapical disease severity. While the dmft index reflects cumulative caries experience, the PAI reflects the radiographic severity of chronic odontogenic infection. By incorporating both parameters, the present study provides a more comprehensive evaluation of the potential systemic consequences of pediatric oral disease than studies relying solely on caries indices [33]. Therefore, the assessment of periapical disease may provide complementary information beyond conventional caries indices for evaluating the systemic inflammatory burden associated with pediatric oral disease. Accordingly, our findings suggest that both caries burden and the severity of chronic odontogenic infection should be considered when investigating the systemic manifestations of ECC in children.

The present study has several strengths. To the best of our knowledge, this is among the first studies to investigate the association between the HALP score and early childhood caries in preschool children. Furthermore, immunonutritional status was assessed not only using the composite HALP score but also through its individual hematological and biochemical components, providing a more comprehensive evaluation of the potential systemic alterations associated with ECC. Restricting the study population to 4-year-old children minimized age-related variability in hematological and biochemical parameters and improved the homogeneity of the study sample. In addition, the integration of clinical (dmft), radiographic (PAI), and laboratory data, together with the use of the highest individual PAI score to represent periapical disease severity, enabled a multidimensional assessment of the potential systemic effects of ECC and constitutes an important methodological strength of the present study.

The present study has several limitations. First, the retrospective single-center comparative design precludes the establishment of causal relationships. Second, inflammatory biomarkers such as ferritin, C-reactive protein, interleukins, and other cytokines were not evaluated, which may have limited a more comprehensive assessment of the systemic inflammatory response. Furthermore, comprehensive nutritional and clinical data, including anthropometric measurements, dietary intake, socioeconomic status, hydration status, and indicators of iron status or recent infection, were unavailable. Consequently, residual confounding cannot be excluded, and serum albumin should not be interpreted as a definitive marker of overall immunonutritional status. Third, the inclusion of only 4-year-old children may limit the generalizability of the findings to other pediatric age groups or to children with less severe caries experience. Another important limitation relates to the selection of the control group. Although both groups consisted of healthy 4-year-old children and laboratory analyses were performed in the same institutional laboratory, participants were recruited from different clinical settings. Therefore, residual selection bias and confounding by indication cannot be excluded, and the observed between-group differences should be interpreted as associations rather than evidence of causality. In addition, the unequal sample sizes between the ECC and control groups should be acknowledged. This imbalance reflects the inherent challenges of retrospectively identifying age-matched, systemically healthy, caries-free children with complete laboratory records and may have reduced the precision of the between-group comparisons. Furthermore, the sample size calculation was performed for the primary case–control comparison and was not specifically designed for the multivariable regression analyses; therefore, the regression findings should be interpreted with appropriate caution. Finally, evidence regarding the clinical applicability of the HALP score in pediatric populations remains limited, and therefore the present findings should be interpreted with appropriate caution. Future multicenter prospective studies including different pediatric age groups, together with comprehensive nutritional and inflammatory assessments, are warranted to validate the clinical utility of the HALP score and to establish pediatric-specific reference or cutoff values.

## 5. Conclusions

Within the limitations of this retrospective comparative study, no statistically significant association was detected between the HALP score and ECC status, caries burden, or periapical disease severity in preschool children. Although albumin levels differed between children with ECC and caries-free controls, these findings were not confirmed in multivariable regression analyses, and the association between hemoglobin and severe periapical disease did not reach statistical significance. Therefore, these observations should be considered hypothesis-generating and require confirmation in prospective studies. These findings underscore the complex relationship between oral and systemic health during early childhood without establishing the clinical utility of the evaluated biomarkers. Future multicenter prospective studies including different pediatric age groups, together with comprehensive nutritional and inflammatory assessments, are warranted to further clarify the role of the HALP score in pediatric populations and to establish age-appropriate reference values. Ultimately, this study contributes to the growing body of evidence exploring the relationship between immunonutritional biomarkers and oral health in preschool children and may help inform future research on biomarker-based risk assessment in pediatric dentistry.

## Figures and Tables

**Table 1:** Distribution of hematological and dental variables.

Variables	Median (IQR)
PAI score	3 (1–5)
dmft	12 (0–20)
Hemoglobin (g/dL)	13.15 (10.8–15.4)
Albumin (g/L)	45 (38–51)
Lymphocyte (×10^9^/L)	3.96 (0.64–10.57)
Platelet (×10^9^/L)	380 (130–677)
HALP score	6.47 (1.66–21.93)

**Table 2:** Spearman’s correlation coefficients (ρ) between the HALP score and dental variables.

Variables	ECC Status	PAI Group	PAI Score	dmft
HALP score	−0.002	−0.018	0.047	−0.040
Variable *p*	0.982	0.845	0.600	0.653

**Table 3:** Comparison of Study Variables According to Periapical Disease Severity.

Variables	Low PAI (n = 88) Median (25–75%)	High PAI (n = 38) Median (25–75%)	*p*
dmft	12 (0–14)	13.5 (12–16)	<0.001
Hemoglobin (g/dL)	13.2 (12.55–13.85)	12.95 (12.5–13.8)	0.303
Albumin (g/L)	45 (43.95–47)	46 (44–47)	0.300
Lymphocyte (×10^9^/L)	3.98 (3.03–4.72)	3.90 (3.1–4.56)	0.987
Platelet (×10^9^/L)	380 (317.5–425)	377.5 (331–419)	0.905
HALP score	6.47 (4.97–7.84)	6.48 (4.57–7.43)	0.844

**Table 4:** Comparison of Study Variables According to ECC Status.

Variables	Case (n = 97) Median (25–75%)	Control (n = 29) Median (25–75%)	*p*
dmft	14 (12–16)	0 (0–0)	<0.001
Hemoglobin (g/dL)	13.2 (12.7–13.9)	12.8 (12–13.6)	0.065
Albumin (g/L)	46 (44–48)	44 (43–44.6)	<0.001
Lymphocyte (×10^9^/L)	3.94 (3.1–4.56)	4.02 (3.24–5.04)	0.624
Platelet (×10^9^/L)	380 (322–437)	380 (310–400)	0.505
HALP score	6.43 (5.02–7.63)	6.8 (4.64–7.79)	0.982

**Table 5:** Negative binomial regression analyses for factors associated with dmft.

**Model 1. HALP score**					
**Variable**	**β**	**SE**	**95% CI**	**Wald χ^2^**	***p***
Intercept	2.460	0.275	1.922–2.998	80.317	<0.001
HALP score	−0.015	0.040	−0.093–0.062	0.149	0.699
**Model 2. Individual hematological parameters**					
**Variable**	**β**	**SE**	**95% CI**	**Wald χ^2^**	***p***
Intercept	−0.819	1.844	−4.433–2.796	0.197	0.657
Hemoglobin	0.045	0.115	−0.181–0.271	0.153	0.696
Albumin	0.058	0.037	−0.013–0.130	2.557	0.110
Lymphocyte	−0.114	0.089	−0.289–0.061	1.642	0.200
Platelet	0.001	0.001	−0.001–0.003	0.595	0.440
**ECC subgroup**					
**Model 3. HALP score**					
**Variable**	**β**	**SE**	**95% CI**	**Wald χ^2^**	***p***
Intercept	2.653	0.316	2.033–3.272	80.427	<0.001
HALP score	−0.005	0.046	−0.095–0.086	0.01	0.919
**Model 4. Individual hematological parameters**					
**Variable**	**β**	**SE**	**95% CI**	**Wald χ^2^**	***p***
Intercept	2.998	2.140	−1.196–7.192	1.963	0.161
Hemoglobin	−0.008	0.130	−0.263–0.246	0.004	0.949
Albumin	−0.010	0.042	−0.092–0.073	0.053	0.818
Lymphocyte	0.014	0.104	−0.191–0.218	0.017	0.897
Platelet	0.000	0.001	−0.002–0.003	0.061	0.806

Negative binomial regression with a log-link function was used. HALP score and its hematological components were analyzed in separate models to avoid mathematical dependency. None of the evaluated variables showed a statistically significant association with dmft. Separate models were fitted for HALP and its hematological components to avoid mathematical dependency.

**Table 6:** Binary logistic regression analysis for factors associated with severe periapical disease (PAI 4–5).

**Variable**	**β**	**SE**	**OR**	**95% CI**	***p***
dmft	0.172	0.046	1.188	1.087–1.299	<0.001
Hemoglobin	−0.532	0.274	0.588	0.344–1.005	0.052
Albumin	0.107	0.088	1.113	0.936–1.323	0.226
Lymphocyte	−0.107	0.214	0.898	0.591–1.366	0.616
Platelet	−0.002	0.003	0.998	0.993–1.004	0.551
Gender (male)	0.125	0.441	1.133	0.477–2.690	0.777
**Model performance**					
**Statistic**			**Value**		
Omnibus χ^2^			24.631		
*p*			<0.001		
Hosmer–Lemeshow χ^2^			4.382		
Hosmer–Lemeshow *p*			0.821		
Cox & Snell R^2^			0.178		
Nagelkerke R^2^			0.251		
Classification accuracy			70.6%		

Binary logistic regression was performed with high PAI (scores 4–5) as the dependent variable. Odds ratios (ORs) are presented with 95% confidence intervals. Sex was coded as 0 = female and 1 = male.

## Data Availability

The datasets generated and/or analyzed during the current study are available from the corresponding author on reasonable request.
